# Cheek tooth repulsion aided by computer-assisted surgery in 16 equids

**DOI:** 10.3389/fvets.2025.1571539

**Published:** 2025-10-08

**Authors:** Micaël D. Klopfenstein Bregger, Mathieu de Preux, Hervé P. Brünisholz, Elke Van der Vekens, Daniela Schweizer, Christoph Koch

**Affiliations:** ^1^Division of Equine Surgery, Swiss Institute of Equine Medicine, Department of Clinical Veterinary Science, Vetsuisse-Faculty, University of Bern, Bern, Switzerland; ^2^Division of Clinical Radiology, Department of Clinical Veterinary Science, Vetsuisse-Faculty, University of Bern, Bern, Switzerland

**Keywords:** cheek tooth, repulsion, computer-assisted surgery, dental surgery, horse

## Abstract

This retrospective case series reports on the use of computer-assisted surgery (CAS) to perform cheek tooth repulsion in 16 equids. Thirteen of the 16 subjects in this case series had a mandibular cheek tooth repulsed, and 3 had a maxillary cheek tooth removed. Surgery was performed on all subjects under general anesthesia, and all but one were placed in lateral recumbency. All cheek teeth were successfully removed by navigated repulsion, except in one case where additional intraoral sectioning was performed. In one horse, a surgical approach through the contralateral nasal conchae was made to facilitate exodontia. This led to considerable hemorrhage and a temporary tracheotomy was performed to ensure airway patency postoperatively. Six subjects needed at least one additional revision surgery to remove either osseous or dental fragments or sequestrated alveolar bone. Outcome was successful in all but one subject, which was euthanized after surgery because a squamous cell carcinoma was diagnosed histologically. The real-time intraoperative guidance provided by CAS allows for controlled and accurate surgical access to targeted dental structures and exodontia of cheek teeth.

## 1 Introduction

In equine patients, cheek tooth extractions are routinely performed to address dental pathologies, including apical infection, fractured, displaced, malerupted/impacted teeth, and polyodontia. In most cases, exodontia is accomplished under local anesthesia via a transoral approach and in the sedated, standing horse. The complex anatomy of the equine head with its adjacent, voluminous nasal conchae and paranasal sinuses, the high density of vascular and neural structures, and the long reserve crowns with compound roots can make exodontia in this species challenging. This is particularly true in cases where the clinical crown is missing, tooth morphology is abnormal, or when remotely located residual fragments are present. Therefore, dental surgery in horses is associated with relatively high complication rates (1–8), which seem to be higher for mandibular (18.1%, 25/138 teeth) than maxillary (9.7%, 28/290 teeth) cheek tooth extractions in one report on post-extraction complications using several extraction techniques (7).

Several alternative dental extraction techniques have been proposed in case of failure of the standard transoral extraction method (9). Dental repulsion is such an alternative technique, with forces applied to the apical region of the affected tooth, with the horse standing or under general anesthesia. However, early reports (1, 3) describe various intraoperative complications in approximately 10% of the cases, including alveolar bone fracture. Postoperative complications such as infection of an adjacent tooth, bone sequestration, chronic sinusitis, or sloughing of the skin-flap occurred in approximately 40% of the cases, and an additional surgical procedure was necessary in 20% (1, 3). Therefore, in modern equine dentistry, dental repulsion techniques are commonly avoided and, in cases where transoral extraction is not possible or cannot be completed, less invasive alternatives are preferred. Recently, however, it has been proposed that the use of smaller diameter pins reduces the risk of complications resulting from dental repulsion, such as the formation of orocutaneous or orosinusoidal fistula (10). On the other hand, resorting to smaller pins may increase the risk of getting the pin entrapped between the tooth and alveolar bone, which can lead to fractures (10).

Regardless of the technique applied, intraoperative imaging modalities are crucial for adequate intraoperative orientation and to avoid inadvertent iatrogenic damage to nearby anatomic structures during complicated exodontia (9, 11–13). With the exception of making a large bone flap to approach maxillary cheek teeth, the surgical orientation during repulsion relies almost exclusively on repeated intraoperative radiographs to identify the apical region of the tooth to be extracted and to align the dental punch with its long axis (1).

The introduction of advanced imaging modalities, including computed tomography (CT), launched the development of computer-assisted surgery (CAS) to improve surgical planning and intraoperative orientation for surgical interventions. CAS is particularly useful in surgeries where exact instrumentation and multiplanar orientation are indispensable, and it facilitates minimally invasive approaches, aiming to decrease collateral damage and improve the overall outcome (14). Ideally, a three-dimensional (3D) image data set of the anatomical region of interest is acquired preoperatively and used for complementary diagnostics and planning as needed. Moreover, tracking systems are used to locate selected navigated surgical instruments in relation to the anatomy. Most navigation systems used for CAS of the head operate with optical or electromagnetic tracking systems (15). Intraoperatively, virtual images are displayed to the surgeon to simultaneously provide real-time information on the position and orientation of both anatomy and navigated instruments.

Hence, early on in its development, CAS has been used specifically for surgical applications involving the head (16). With recent technological advances, CAS has become an integral part of the clinical routine in ear, nose and throat, and cranio-maxillofacial surgery in humans (17, 18). To the best of our knowledge, in equids, the use of CAS has so far been limited to orthopedic surgery applications (19–27) and neuronavigation (28–31) in experimental and clinical settings. Specific reports of clinical applications of CAS in horses involving the head are limited to removing an ectopic tooth at the base of the skull and a navigated surgical approach to access a pituitary macroadenoma in a mare (32, 33). The aim of this study is to describe potential indications, technical procedural details, and complications of dental repulsions aided by CAS in selected cases.

## 2 Materials and methods

Horses referred to the ISME Equine Clinic Bern between December 2015 and April 2023, that underwent maxillary or mandibular CAS repulsion were included in this study. Data obtained from the medical records comprised age, sex, breed, presenting complaint, diagnosis, surgical technique, complications, and information pertaining to the perioperative case management.

### 2.1 Preoperative patient preparation

All subjects received benzylpenicillin sodium (30,000 IU/kg IV, Penicillin Natrium Streuli for animal use only; Streuli Pharma AG, Uznach, Switzerland), gentamicin sulfate (6.6 mg/kg IV, Pargenta-50 for animal use only; Dr. E. Graeub AG, Bern, Switzerland), and flunixin meglumine (1.1 mg/kg IV Vetaflumex for animal use only; Covetrus, Lyssach, Switzerland) approximately one hour prior to surgery. Subjects were premedicated with acepromazine (0.03 mg/kg IM, Prequillan for animal use only; Fatro SpA, Ozzano dell’Emilia, Italy) 20 min prior to induction and sedation with romifidine (0.04 mg/kg IV, Sedivet for animal use only; Boehringer Ingelheim, Basel, Switzerland) and levomethadone (0.05 mg/kg IV, L-Polamivet for animal use only; MSD Animal Health GmbH, Lucerne, Switzerland). General anesthesia was induced with a combination of ketamine (2.5 mg/kg IV, Ketasol-100 for animal use only; Dr. E. Graeub AG, Bern, Switzerland) and diazepam (0.05 mg/kg IV, Valium; Roche Pharma AG, Basel, Switzerland) and maintained with isoflurane. The anesthetized subjects were positioned in lateral recumbency with the affected side up, except case 16, where dorsal recumbency with a tilted head was chosen. Aseptic preparation of the surgical field and draping were performed routinely. All subjects were intubated nasally to allow unrestricted access to the oral cavity for the surgical procedure.

**Figure 1:**
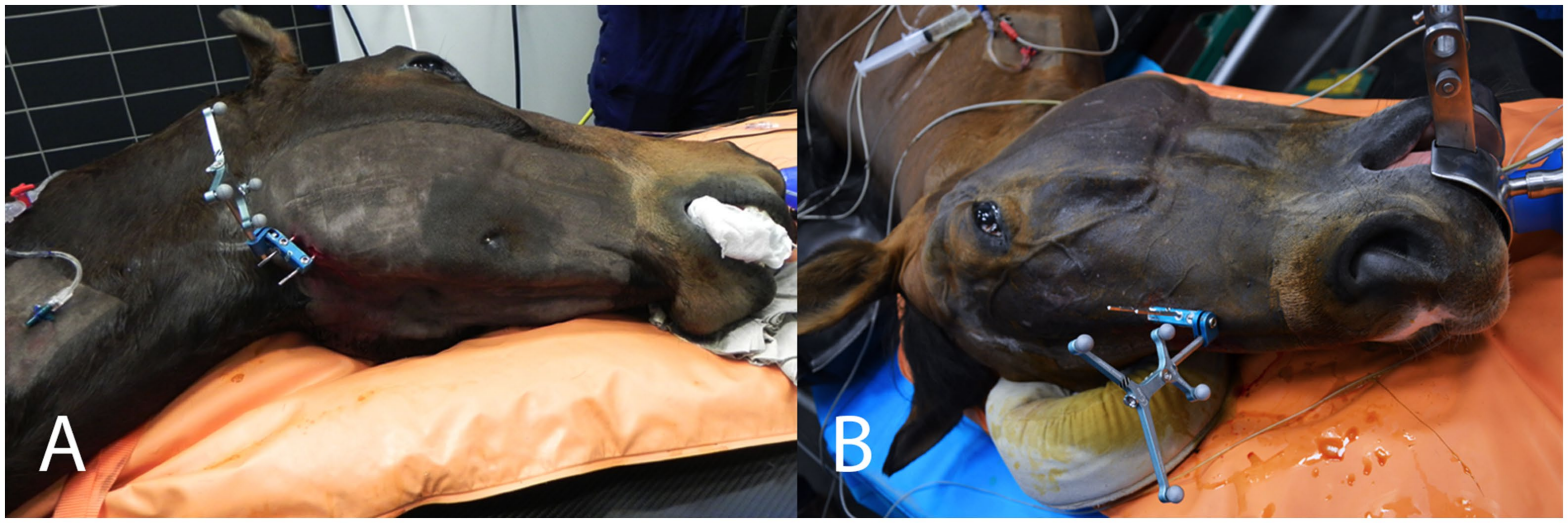
**(A)** Horse in left lateral recumbency for a navigated repulsion of tooth 409 (case 3). The patient tracker is anchored to the exposed, flat, ventral surface of the angle of the mandible. **(B)** Horse in right lateral recumbency for a navigated repulsion of the maxillary cheek tooth 210 (case 14). The patient tracker is anchored to the nasal bone with two 3.2 mm Schanz pins.

For mandibular cheek tooth repulsions (cases 1–13), the patient tracker was anchored with two self-tapping 3.2 mm Schanz pins drilled into the flat ventral surface of the angle of the uppermost mandible (Figure 1A). The patient tracker serves as a fixed reference point for the optical tracking system and is not supposed to move in relation to the structure to be operated on. For maxillary cheek tooth repulsions (cases 14–15), the patient tracker was attached with two self-tapping pins either in the uppermost nasal bone at the level of the naso-incisive notch or in the facial crest (Figure 1B).

#### 2.1.1 Preoperative imaging and planning

**Figure 2:**
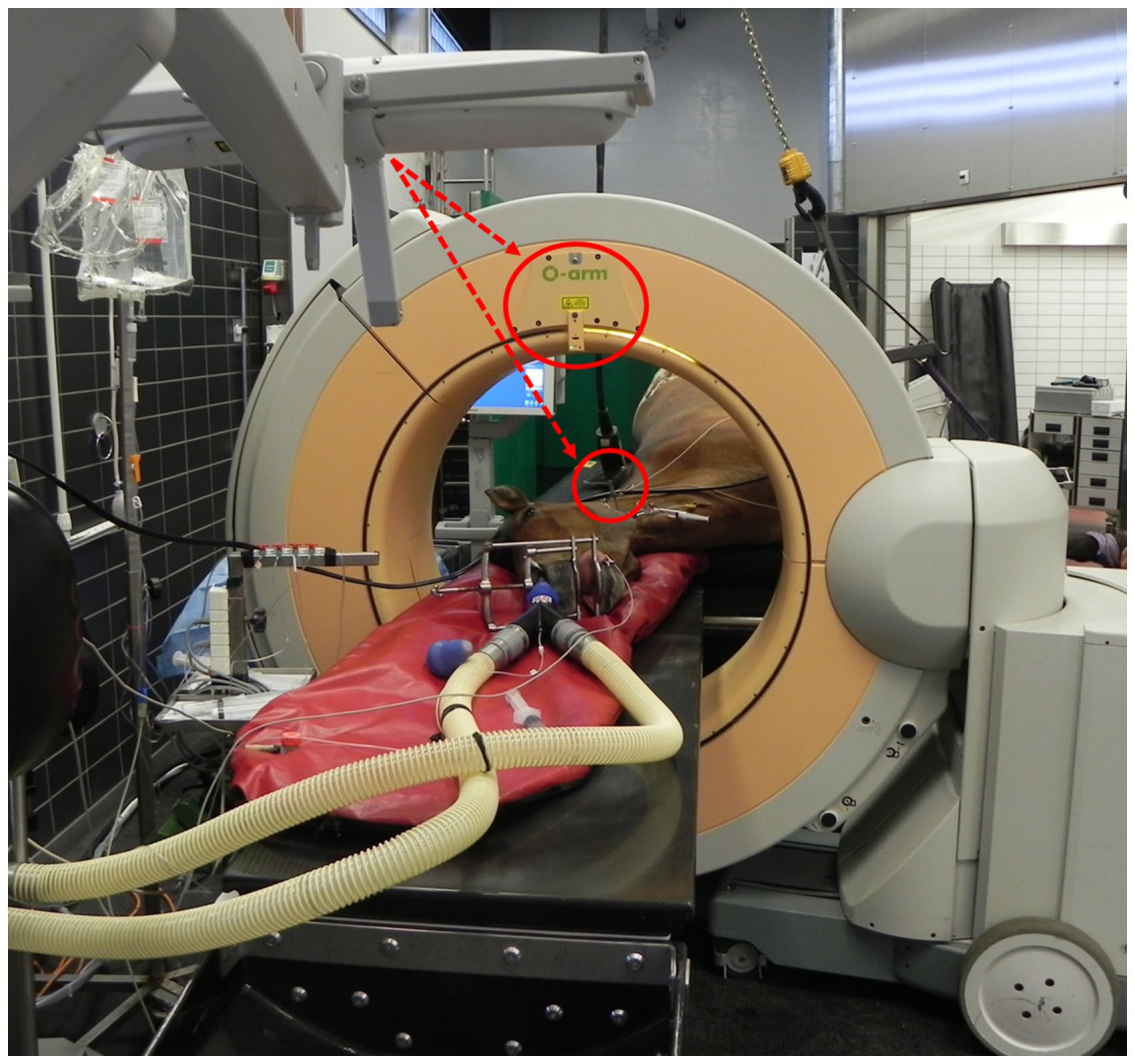
Overview of a computer-assisted surgery within the preoperative room immediately following preoperative image acquisition (case 4). All necessary equipment for imaging, planning and navigation is shown, i.e., the cone beam computed tomographic scanner (O-arm) coupled with the StealthStation navigation system (both Medtronic, only camera visible on image). The horse’s head is placed on a carbon table and the patient tracker (red circle) is anchored to the left mandible. Please note that the beacon of the camera is oriented to simultaneously detect both patient- and gantry-tracker (dashed red lines).

A mobile cone beam CT (CBCT) (O-arm 1 or 2 since 2023, Medtronic, Louisville, Colorado) was used for pre- and intraoperative 2D (fluoroscopic) and cross-sectional imaging. To complete the fully functional CBCT-based CAS setup, the CBCT was used in conjunction with the StealthStation SS7 or SS8 (since 2023, cases 13, 15, and 16; Medtronic) navigation system (Figure 2). A carbon fiber table (Opera Swing, General Medical Merate S. P. A., Seriate, Italy) was used to support the subject’s head and neck for imaging and surgery (Figure 2).

Following patient preparation and tracker fixation, a preoperative CBCT scan was acquired. First, adequate positioning of the anatomical region of interest in the center of the gantry was confirmed with two orthogonal 2D fluoroscopic projections. Next, the camera of the navigation system was oriented to simultaneously detect the tracker of the CBCT gantry and the patient tracker (Figure 2). A standard acquisition, i.e., 391 projections during one tube rotation using an exposure of 120 kV and 64 mAs, reconstructed in 192 isovolumetric images, was performed. If increased detail was desired, a high-definition scan was acquired based on 745 projections, which doubled the acquisition time from 13 to 26 s. During pre- and intraoperative imaging, all personnel left the room to avoid radiation exposure.

**Figure 3:**
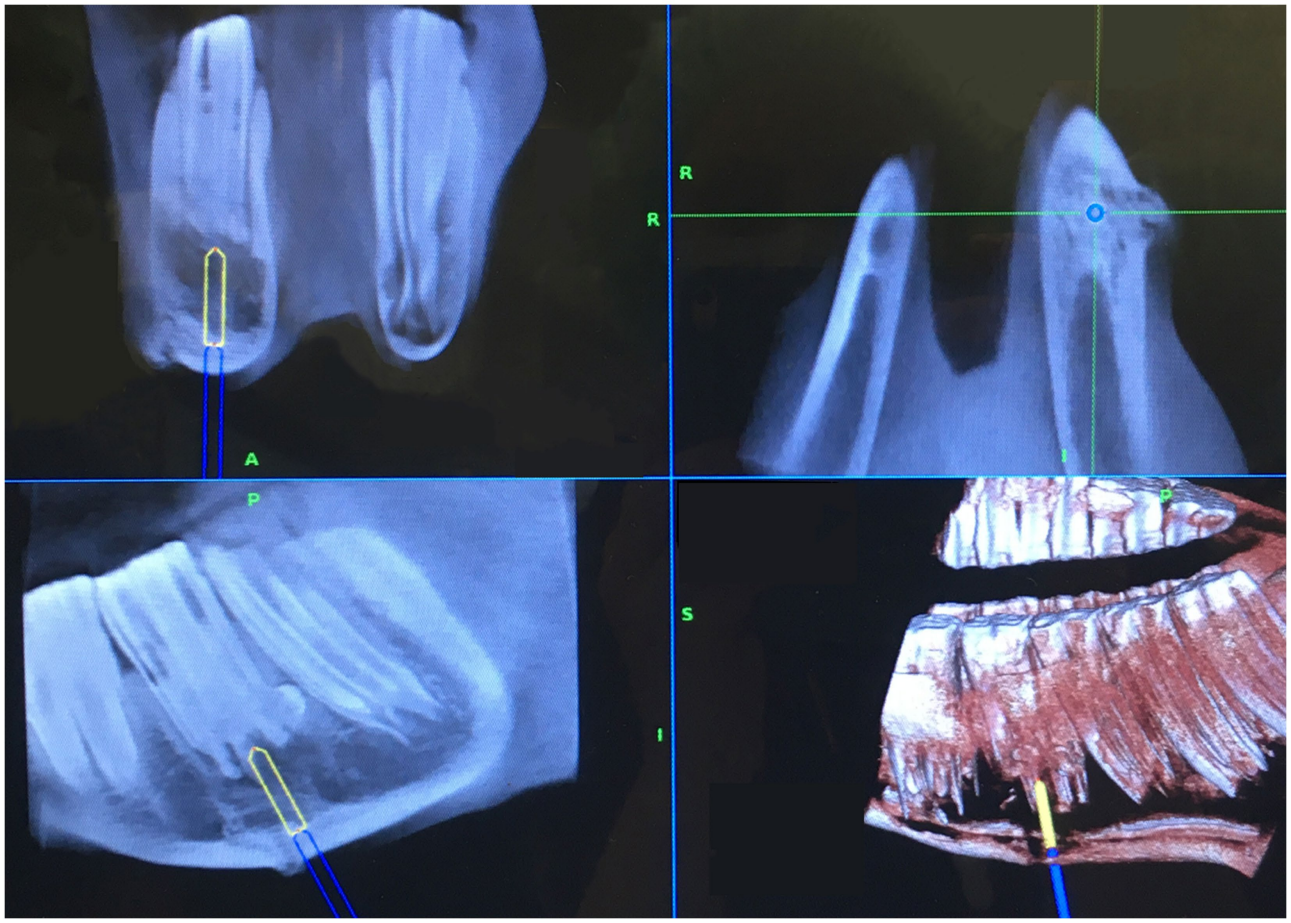
Screen shot of the monitor displaying the preoperative plan to the operating surgeon in different planes (Cranial Software by Medtronic) for a computer-assisted surgical repulsion. In this case (case 7, tooth 308), the step of making an osteotomy through the ventral mandibular cortex is shown. Note: the cheek tooth 308 shows obvious bullous apical enlargement of its distal root. The position and orientation of the navigated drill are shown as a blue cylinder that has already passed the ventral cortex, with a projected trajectory toward the affected tooth (yellow cylinder/line).

**Figure 4:**
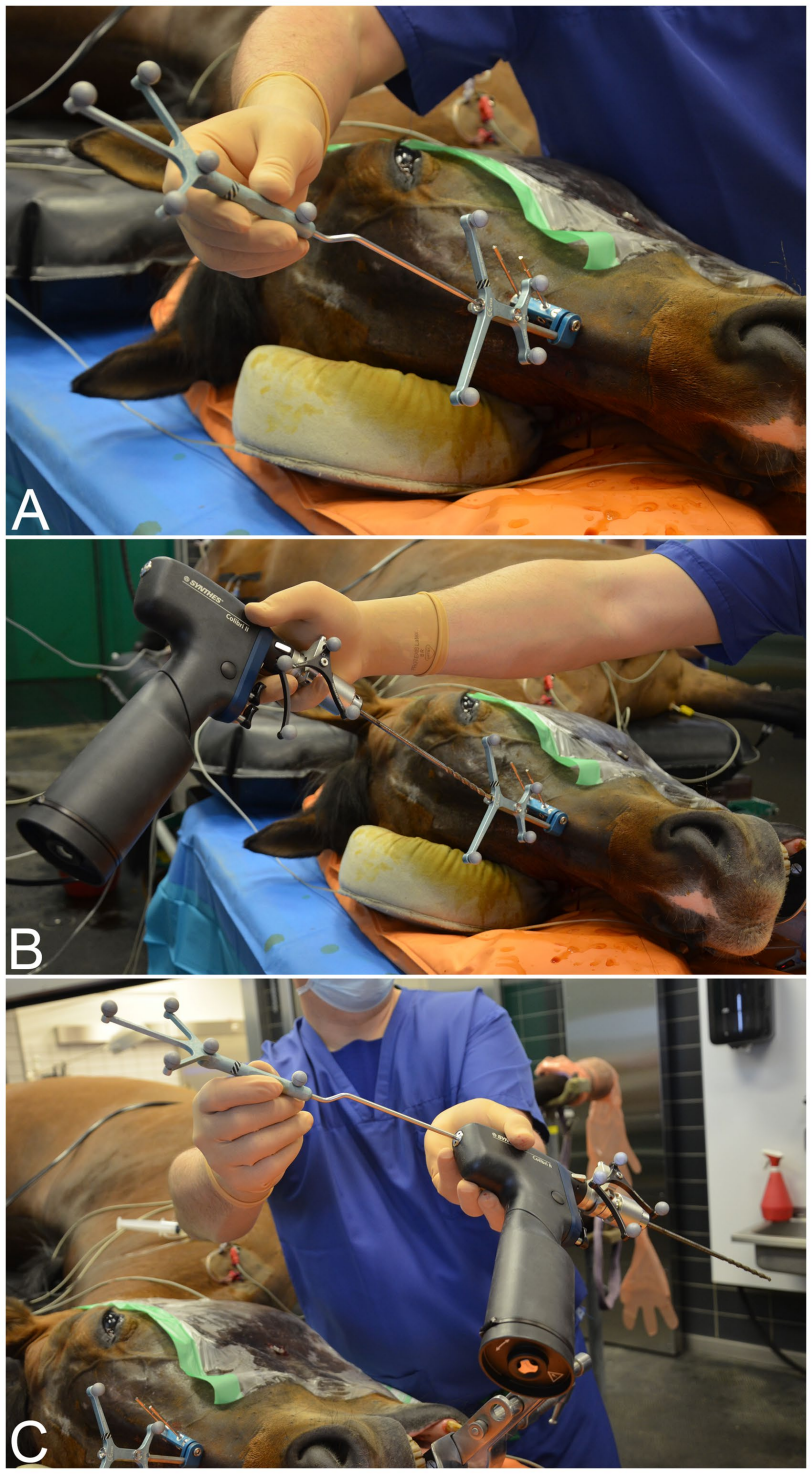
Key elements of a computer-assisted procedure are shown (case 14). Patient registration: following image acquisition, the surgeon contacts the patient tracker with the navigated pointer **(A)**. This step is necessary to initiate any computer-assisted procedure using an optical tracking system, as it links the virtual data set with the real surgical anatomy. **(B)** Instrument calibration: this includes a sequence of consecutive steps to identify the plane, tip **(B)**, and long axis **(C)** of the instrument.

The acquired CBCT dataset was automatically transferred to the navigation system. The surgeon and a radiologist then assessed the CT images for adequate image quality and surgical planning purposes. Preoperative surgical planning was performed with the SS7 or SS8 Cranial, or Spine and Trauma Software (Medtronic) (Figure 3). Prior to any surgical manipulation, the CBCT was moved away from the surgical area to give unrestricted access to the surgical site. To initiate the navigated procedure, the surgeon contacted the patient tracker with the navigated pointer, thus linking the subject’s real anatomy with its virtual image, a process called patient registration (Figure 4A).

#### 2.1.2 Preoperative preparation of navigated instruments

For cases that required navigated drilling, a battery-powered surgical drill (Colibri II, DePuy Synthes, Oberdorf, Switzerland, or Hilti, Schaan, Liechtenstein) was mounted with a tracker (SureTrak II Universal Tracker, Large Passive Fighter, 961–581, Medtronic) on the instrument shaft. Other surgical instruments that were navigated for CAS repulsions included dental punches and a high-speed surgical drill (Midas Rex MR8, Medtronic). In the final step of preparing for intraoperative guidance, the navigated instrument had to be calibrated (Figures 4B,C).

### 2.2 Surgical procedures

#### 2.2.1 CAS repulsion of mandibular cheek teeth

**Figure 5:**
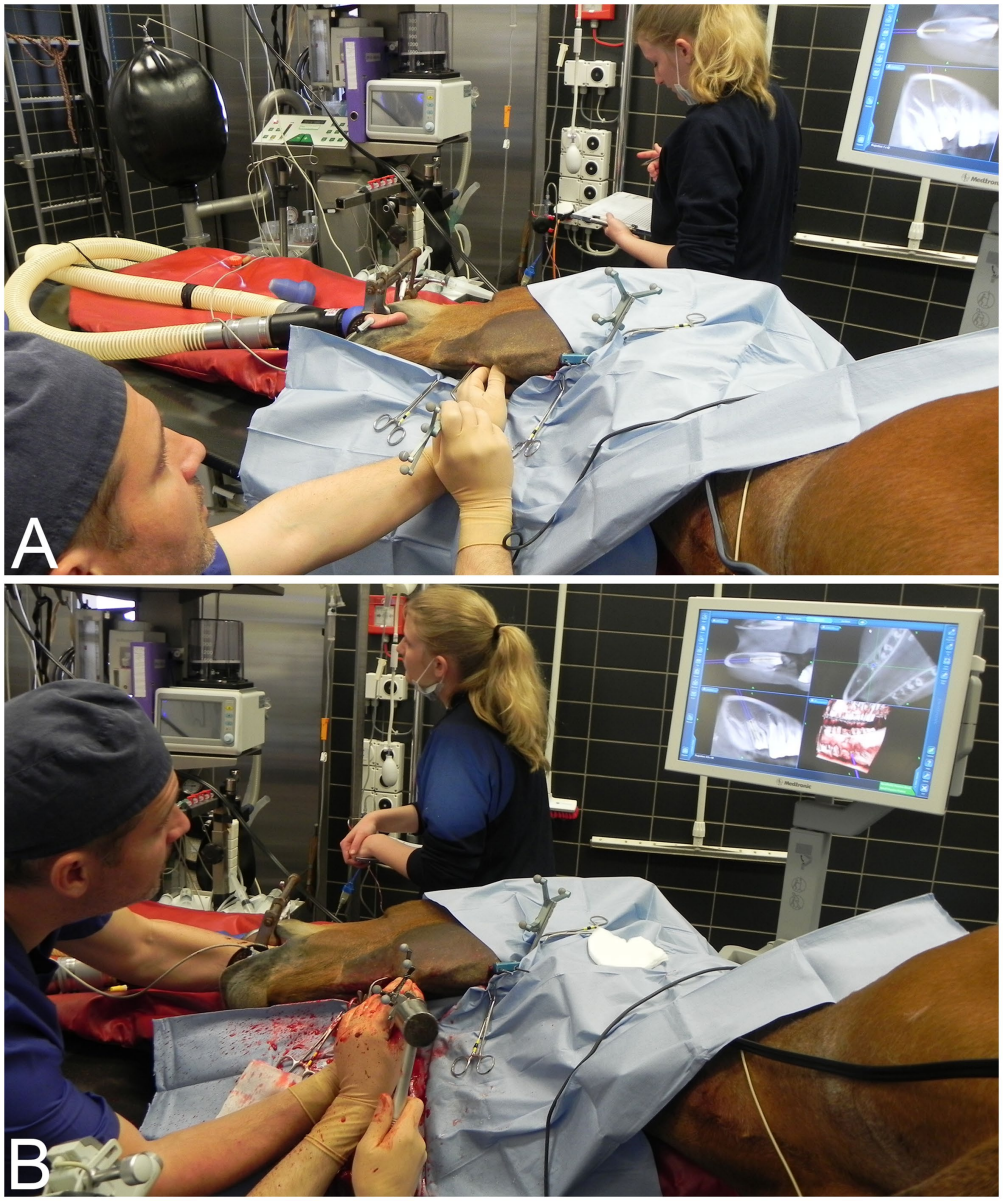
Horse in right lateral recumbency for a computer-assisted surgical repulsion of tooth 309 (case 4). **(A)** The appropriate site for skin incision is determined with the navigated pointer. **(B)** The tooth is repulsed with the navigated dental punch, under real-time orientation and depth control.

**Figure 6:**
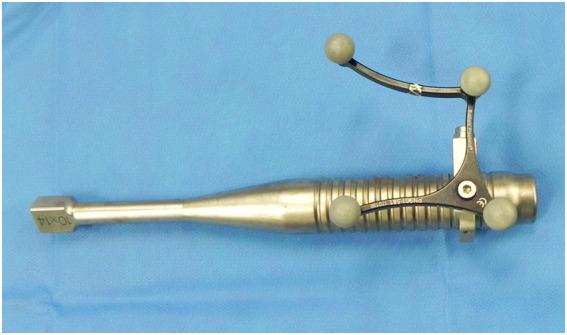
Picture of the navigated rectangular dental punch (10 x 14 mm).

Following patient registration, the ideal site for fenestration of the overlying bone to approach the apical region of the affected cheek tooth was assessed on multiplanar reconstructions of the volumetric scan. The appropriate position for the skin incision was determined with the navigated pointer (Figure 5A) followed by a skin incision reaching the surface of the bone. Whenever a fistulous tract was present that matched the appropriate osteotomy site as assessed by the navigated pointer, a fusiform incision was made around the fistula. The apex of the cheek tooth was exposed by removing the overlying bone. When small cylindrical dental punches were planned to be used for repulsion, drills of increasing diameter were used to expose the apex. Alternatively, to create larger bone fenestrations that accommodate dental punches with a rectangular head (in most of the cases 10 × 14 mm, Figure 6), a 3.5 mm drill was used to penetrate the mandible at the designated corners of the bone window before connecting the corner holes with an oscillating saw or using a high-speed surgical drill. Once the osteotomy was large enough to accommodate a dental punch of appropriate size, the tooth was repulsed with the navigated dental punch (Figures 5B, 6) under real-time orientation and depth control. The virtual projection of the navigated punch helped to correctly align it with the long axis of the tooth while repulsion forces were applied to the apical region. Simultaneously, orad advancement of the targeted cheek tooth during repulsion was monitored manually within the oral cavity.

In cases where, based on the preoperative CT dataset, repulsion was expected to be complicated by an apical malformation and enlargement, a high-speed surgical drill was used to remove excessive dental tissue in the apical region that would impede repulsion or considerably increase the forces necessary for repulsion.

#### 2.2.2 CAS repulsions of maxillary cheek teeth

**Figure 7:**
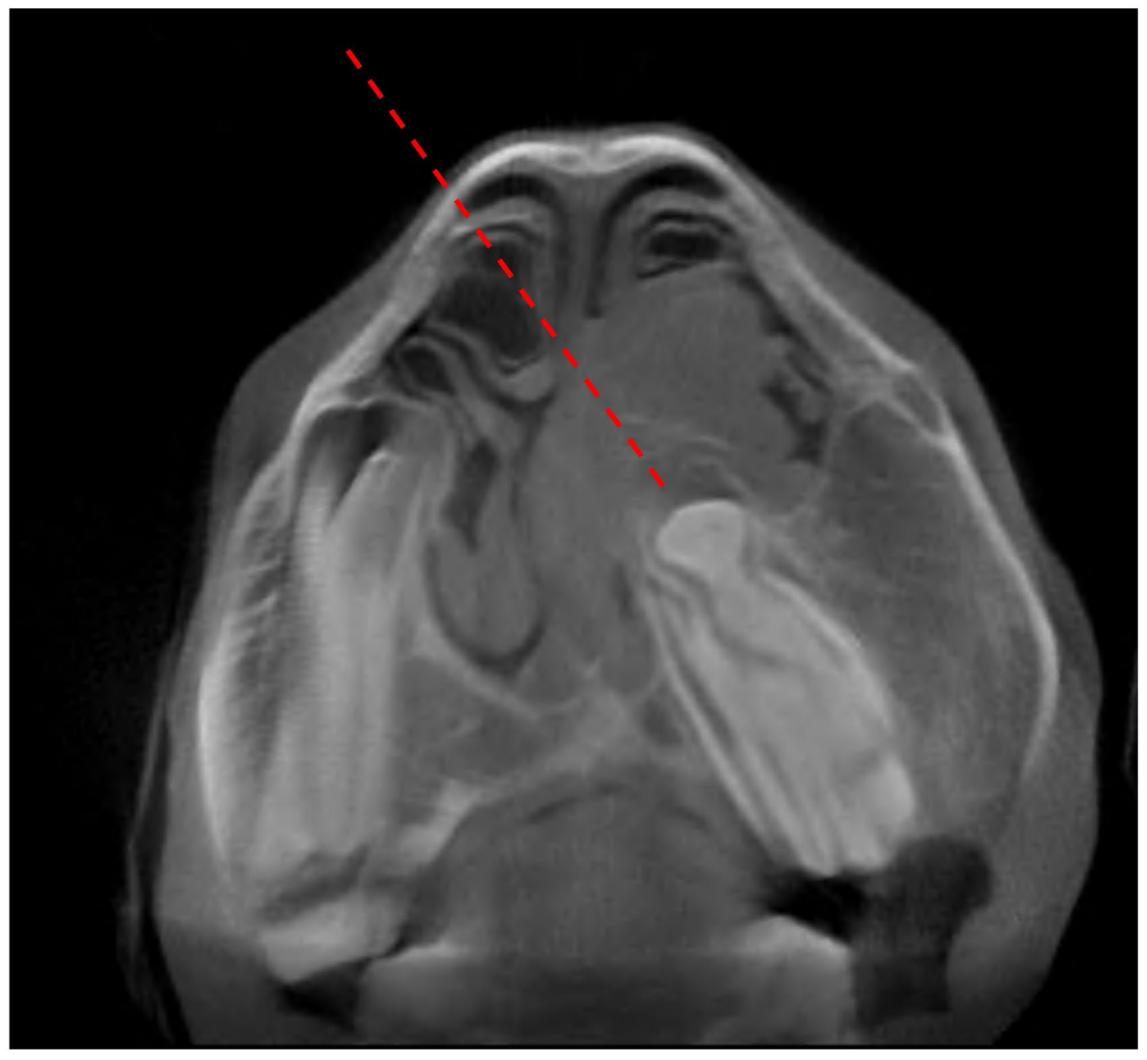
Transverse preoperative cone beam computed tomographic image through tooth 208 (case 16). Note the obliquely orientated and displaced cheek tooth with its tooth roots obstructing the left nasal passage. There is a periapical space occupying mass causing deformation of the left ventral and dorsal conchae. The dashed line represents the direction of repulsion through the right dorsal concha.

For cases 14 and 15, a conchofrontal or rostral maxillary approach, respectively, was made for CAS repulsion. After locating the ideal site for creating the approach with the navigated pointer, a skin incision to the surface of the bone was performed, followed by wound retraction with Gelpi retractors before a 25 mm manual Galt trephine was used to fenestrate the bone and gain a direct approach to the apical region of the affected maxillary cheek tooth. Subsequently, the targeted maxillary cheek tooth or tooth fragments were repulsed with a navigated 8 mm cylindrical dental punch under real-time orientation and depth control. In case 16, an osteotomy was performed in the contralateral right nasal bone to gain access through the right dorsal nasal concha to the displaced obliquely orientated cheek tooth 208 (Figure 7). Navigated cylindrical dental punches of different diameters were used to complete exodontia.

#### 2.2.3 Intraoperative imaging, management of the empty alveolus, and wound closure

Additional intraoperative CBCT scans were acquired in cases where orad advancement of the targeted dental structure was absent or deemed insufficient while applying the repulsion forces. Moreover, CBCT scans were repeated intraoperatively to confirm complete removal of the repulsed cheek tooth and the absence of any residual dental or osseous fragments as deemed necessary.

After successful repulsion, the extracted tooth or dental fragments and the alveolus were inspected visually and digitally for integrity or any residual material, respectively. If necessary, the alveolus was again curetted and lavaged as needed before it was sealed with a silicone plug (3 M Express™ Putty Soft, Uniserv AG, St.-Gallen, Switzerland). Following the mandibular CAS repulsions, the skin incisions were partially closed with simple interrupted sutures using non-absorbable 2–0 suture material (Prolene, ETHICON LLC, Johnson & Johnson, Zug, Switzerland), leaving an opening at the most dependent part of the incision to allow for drainage. Following maxillary CAS repulsions, the skin incisions over the conchofrontal or maxillary trephinations were partially closed, allowing for the placement of a Foley catheter and repeated postoperative lavage of the involved paranasal sinuses. In all cases, the two stab incisions used to anchor the patient tracker were closed with simple interrupted sutures using non-absorbable 2–0 monofilament suture material (Prolene, ETHICON LLC, Johnson & Johnson, Zug, Switzerland).

### 2.3 Postoperative case management

All subjects received benzylpenicillin sodium (30,000 IU/kg IV) postoperatively. Depending on the lesion treated in each case, antimicrobial and non-steroidal anti-inflammatory medication were continued for variable durations at the discretion of the clinician, and antimicrobial medication was adapted based on results from bacterial culturing and sensitivity testing. In horses registered as companion animals, phenylbutazone (2.2 mg/kg BID PO) was the non-steroidal anti-inflammatory agent of choice, whereas horses registered as food producing animals routinely received flunixin meglumine (1.1 mg/kg SID or BID PO, Cronyxin, Grovet, Holland).

### 2.4 Assessment of surgical complications and outcome

All surgery reports were reviewed for mention of any intraoperative complications. After hospital discharge, all horses were subjected to clinical control examinations and treatments by the referring veterinarian or at our institution at variable time intervals. Additional follow-up information regarding postoperative complications and outcomes was obtained via telephone interview with the owners. Cases that required revision surgery(ies) other than a routine alveolar plug shortening, or cases with delayed healing (>8 weeks for complete epithelization of the surgical site) were defined as having a postoperative complication.

## 3 Results

### 3.1 Case overview

Sixteen subjects were identified, including 9 mares, 2 stallions, and 5 geldings with a median age of 7.5 years (range 3–20). The breed distribution was eight Warmbloods, two Franches-Montagnes horses, two Standardbreds, one Quarter Horse, one Appaloosa, one Haflinger, and one Shetland pony.

Thirteen (82%) of the 16 CAS repulsions were performed on mandibular teeth, and 3 (18%) on maxillary teeth.

### 3.2 Initial clinical findings and diagnostic imaging findings

Twelve of the 16 subjects had a mandibular (*n* = 11) or facial (*n* = 1) swelling at admission, with seven of these having an external draining tract (fistula), all on the ventral/ventrolateral aspect of the mandible (Table 1).

**Table 1:** Case details for cases subjected to CAS repulsion (*removal of fragments and curettage).

Case Nr	Age	Triadan position	External swelling	Draining tract	Tooth deformity	Underlying pathologies	Attempted transoral extraction	Decision-making factors for CAS repulsion	Intraoperative complication(s)	Number of additional surgery(ies)*
1	20	411	Yes	Yes	No	Apical infection bone lysis	No	Pain/lack of compliance→ inability to place mouth gag and restricted access to caudal oral cavity, desired access for bone debridement	None	-
2	4	308	Yes	Yes	No	Apical infection 308 intraalveolar crown and root fracture 308	No	Intraalveolar tooth fracture and long reserve crown, concurrent horizontal fracture of the reserve crown of adjacent 307 (for this reason, standing transoral tooth loosening and extraction were not attempted)	Slab fracture of 309	1
3	3	409	Yes	Yes	No	Apical infection	Yes (standing)	Failed transoral extraction, loss of clinical crown, reserve crown wedged between adjacent teeth	None	-
4	14	309	No	No	Yes	Fractured clinical crown pulpitis	No	Missing clinical crown and apical malformation	None	-
5	13	408	Yes	No	No	Apical infection	No	Reasons are unclear and can no longer be reconstructed	None	-
6	8	409	No	No	No	Fractured clinical crown pulpitis	No	Friable, fractured clinical crown	None	-
7	5	308	Yes	No	Yes	Apical infection	No	Apical malformation and enlargement	None	-
8	16	309	Yes	Yes	No	fractured clinical crown pulpitis mandibular sequestrum	No	fractured clinical crown and triangular-shaped mandibular sequestrum	None	-
9	6	308	Yes	Yes	Yes	Apical infection	Yes (under same g/a)	Failed transoral extraction, loss of clinical crown, long reserve crown, apical malformation and enlargement, restricted access in a small pony	Focal disruption of medial alveolar bone	-
10	8	307	Yes	Yes	No	Fractured tooth root apical infection (suspected concurrent apical infection of adjacent 306)	No	Fractured tooth root	None	2 (incl. extraction of adjacent 306)
11	7	408	Yes	Yes	Yes	Fractured clinical crown pulpitis	No	Friable and incomplete clinical crown, apical malformation	None	5
12	6	409	Yes	No	No	Fractured clinical crown pulpitis	No	Friable, fractured clinical crown	None	1
13	15	408	Yes	No	Yes	Apical infection	No	Friable clinical crown, apical malformation and enlargement	Intraoral sectioning required	2
14	14	210	No	No	No	Apical infection sinusitis with empyema of the left ventral conchal bulla	Yes (standing)	Failed transoral extraction, loss of clinical crown, lack of compliance, failed navigated mitse, possibility to perform a cas-guided direct extra-nasal approach to the left ventral conchal bulla	None	-
15	6	109	No	No	No	Apical infection sinusitis orosinusoidal fistula	Yes (standing, incl. mitse)	Failed transoral extraction incl. mitse, loss of clinical crown, failed navigated mitse	Focal disruption of lateral alveolar bone	-
16	3	208	Yes	No	No	Apical infection obstructed left nasal passages	No	Displaced, unerupted cheek tooth, failed navigated mitse	Bleeding	2

CAS, computer-assisted surgery; G/A, general anesthesia; MITSE, minimally invasive transbuccal screw extraction.

In case 1, swelling in the region of the masseter muscle was noticed 6 weeks before admission, and an abscess was opened and drained at the most ventral aspect 2 weeks before admission. An orocutaneous fistula could be confirmed after the initial exam. The horse showed poor compliance with the oral examination and reacted painfully to any manipulations of the affected region. Besides the obvious signs of an apical infection of tooth 411, associated bone lysis was visible on CBCT images.

Case 16 was initially presented for a facial swelling rostral to the facial crest. Upon initial presentation, 1 year prior to CAS-repulsion of tooth 208, an abscess associated with the deciduous tooth 608 was drained, and the permanent tooth 208 was found displaced toward palatinal and rostral but not obstructing the nasal passages.

**Figure 8:**
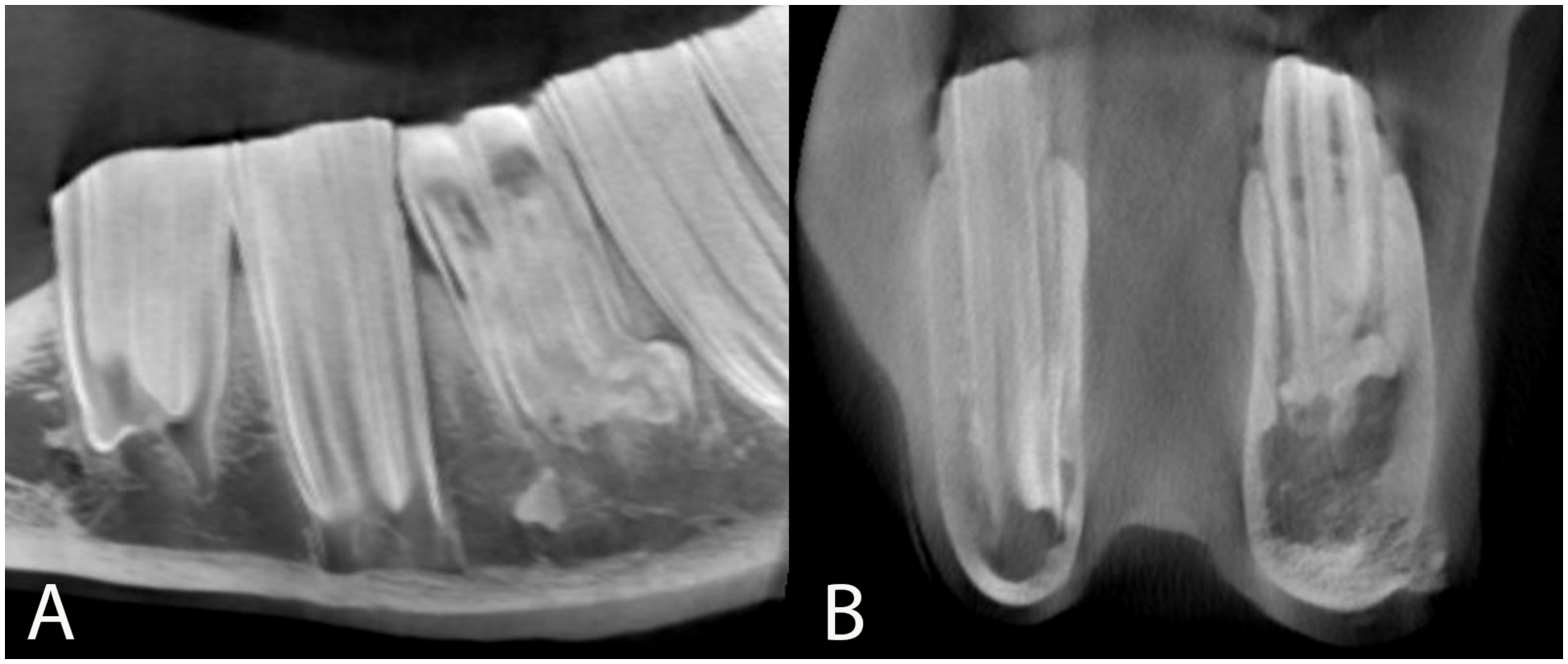
Sagittal **(A)** and transverse **(B)** multiplanar reconstructions of a preoperative cone beam computed tomographic scan through tooth 308 (case 7) also shown in Figure 3. The apical aspects of this tooth are malformed, showing a distal bulbous enlargement and a lingual and buccal bulbous widening on its mesial aspect. **(A)** Note the decreased coronal width of 308 at its occlusive surface and the mild horizontal alveolar bone loss mesial to 308. An isolated fractured root fragment is visible on the mesial aspect of the roots of tooth 308. Additional computed tomographic findings at the level of this tooth are diffuse endosteal thickening of the ventrolateral left mandibular cortex and mild adjacent soft tissue swelling. This cortex shows a focal cortical interruption as well as periosteal reaction. The mesial and distal infundibula of tooth 308 are hypoattenuating and widened, compatible with infundibular hypoplasia. **(B)** Note the close contact between the bone and the apical region of the affected tooth, while the periodontal space is widened apical to this malformation on the buccal aspect.

Apical malformation and enlargement (including peripheral cementum deposition) was found in five subjects to varying extents, all of whom had a mandibular cheek tooth affected (Table 1; Figure 8).

The diagnosed underlying disease(s) that prompted exodontia of these 16 cheek teeth were apical infection (*n* = 11), with or without fractured tooth roots and/or displacement, and endodontic diseases (*n* = 5), with or without partial fractures and pulpitis.

In case 10, CBCT imaging revealed a fistulous tract that extended not only to the infected and subsequently repulsed 307, but also to the distal root of the adjacent 306. Because the root of 306 itself was radiographically not clearly affected, it was decided not to remove it at that time.

In case 15, cheek tooth 108 had been inadvertently penetrated with a 5 mm drill when attempting to extract the 109 using a standing MITSE technique. At the time of the control examination, 3 months after surgery, there was no evidence of an ongoing infectious process involving tooth 108, with a completely healed alveolus of the adjacent repulsed cheek tooth.

### 3.3 Surgical techniques, intraoperative complications, intraoperative imaging, and short-term outcome

#### 3.3.1 Surgical techniques

A transoral approach was initially attempted in four subjects (cases 3, 9, 14, and 15), either in the standing horse or under the same general anesthesia as for the CAS repulsion (Table 1). Gingival elevation, spreading, tooth loosening with extraction forceps, and extraction attempts with a fulcrum were performed routinely. All 4 attempts led to the loss of the entire clinical crown. In case 15, an attempted standing MITSE was also unsuccessful.

In the remaining 12 cases, the treating surgeon proceeded directly with CAS repulsion under general anesthesia. These decisions were based on findings from the oral cavity examination, such as missing or fractured clinical crowns, or radiological findings, such as tooth deformities like apical malformation and enlargement. Other considerations that prompted surgeons to opt directly for CAS repulsion included the patient age, as long reserve crowns are present in younger patients, restricted accessibility of the lesions through the oral cavity, and/or anticipated lack of patient compliance with a standing procedure (Table 1).

In all three CAS repulsions of maxillary cheek teeth, a MITSE technique was attempted once the horse was under general anesthesia (second attempt in case 15). Intradental drilling was performed under CAS navigation, which allowed for precise screw positioning. However, the thread within the tooth stripped in all cases when applying extraction forces. This was most likely caused by insufficient mineral density and limited tooth loosening in the unerupted tooth (case 16) or consequential to advanced dental disease and decay in the other two cases (14 and 15).

The decision-making factors to perform a CAS repulsion in these cases are summarized in Table 1.

In two out of five subjects with a tooth deformity diagnosed radiologically, the apical malformation and enlargement were judged severe based on CBCT images (cases 7 and 13) (Figure 8). Dental substance was removed with a high-speed surgical drill under CAS navigation once the approach to the apex of the tooth had been established. In case 13, an intraoperative scan was made to reassess the situation because the tooth failed to advance despite considerable forces being applied with the mallet. Even after additional removal of dental substance in the apical region, the repulsion attempts were abandoned because of concerns of fracturing the mandible, and the tooth was sectioned in the transverse plane intraorally with a carbide burr mounted on the IC300 (PZ Technik, Hachenburg, Germany) and removed in two parts.

#### 3.3.2 Intraoperative complications

In case 2, a small slab fracture was created on the mesiobuccal aspect above the gingival level during CAS repulsion on the adjacent tooth (309). This presumably resulted as a consequence of two particularities of this case: Tooth loosening was never performed, due to concerns of inflicting further damage to the fractured adjacent tooth (307), and convergence with the clinical crown of the distally adjacent tooth (309). In cases 9 and 15, a focal disruption of the alveolar bone was noted on the CBCT images after completion of surgery. This was clearly explained by the entrapment of the small diameter dental punch (8 mm) between the medial cortical bone and the targeted tooth in case 9.

The surgical approach through the contralateral right nasal conchae and the nasal septum in case 16 led to severe intraoperative bleeding, which ceased after a tamponade was placed in the right nasal passage. To maintain a patent airway in the postoperative phase, a temporary tracheotomy was performed, and the tracheal tube and tamponade were removed 2 days after surgery.

#### 3.3.3 Intraoperative imaging

Intraoperative control CBCT scans were acquired in 14 of the 16 cases, at the end of surgery to confirm complete removal of the cheek tooth. In case 1, there were remaining small osseous fragments visible on the last scan. In case 8, a sequestrum was still visible in the last intraoperative scan, and it was decided to leave it in place due to its anatomical localization and its extent and shape. In case 16, an intraoperative CBCT scan was acquired after having changed from one lateral recumbency to the other lateral recumbency to facilitate a contralateral transconchal approach, which required re-positioning of the patient tracker from the left to the right facial crest.

#### 3.3.4 Short-term outcome

All subjects were discharged from the hospital, except case 1, which was euthanized after a squamous cell carcinoma was diagnosed histologically from periodontal tissues collected at the time of the CAS repulsion of the targeted mandibular molar (411). The mean hospitalization time for cases subjected to CAS repulsion was 4.5 days.

### 3.4 Postoperative complications and long-term outcome

Case 9 was euthanized 10 months after surgery due to colic and neurologic symptoms, unrelated to the CAS repulsion, leaving 14 subjects for the assessment of long-term follow-up (> 12 months). At the time of final follow-up, clinical signs indicative of residual disease were absent in all 14 cases. In cases 5 and 6, purulent discharge persisted from the repulsion site at the ventral aspect of the mandible for several (up to 8) weeks but resolved spontaneously without further surgical treatments.

At least one additional surgery (and up to 5) was performed in the standing sedated subject in six cases to remove either bony or dental fragments, or sequestrated alveolar bone (Table 1). The additional interventions that ultimately led to the resolution of clinical signs were performed at 2 (case 2), 15 (case 10), 6 (case 11), 3 (case 12), 4 (case 13), and 1 month (case 16) after CAS repulsion. Interestingly, all of the 6 horses where additional surgeries were performed, presented an external swelling at initial presentation, three of them had an external draining tract, and two of them showed an apical malformation and enlargement visible on CBCT images. In case 10, the discharge on the ventral aspect of the mandible persisted until the adjacent tooth (306) was extracted by the private veterinarian 15 months later because of progressive changes in the apical region of that tooth.

## 4 Discussion

The present case series provides a first description of the clinical application of CAS for equine dental surgery. The experiences and case details described here assert that CBCT-based navigation and optical tracking systems can be an integral part of the case management for selected dentistry cases. The technology proved practical and reliable in facilitating challenging exodontia in equids. At the authors’ referral practice, CAS is used regularly to assist dental and paranasal sinus surgeries, which demand state-of-the-art intraoperative image guidance. This report focused on challenging dental extractions, where a transoral approach failed or was no longer possible, or where the benefit of CAS repulsion under general anesthesia over more conventional means in the standing sedated subject was deemed substantial. The 16 cases presented herein comprise approximately 6% of all cheek tooth extractions (data not shown) performed at our institution during the study period. The decision to opt for CAS repulsion over alternative techniques was reached by the operating surgeons on a case-by-case basis. Besides surgeon’s preference, or the fact that alternatives (like MITSE) had failed, the most important factors driving this decision, included the absence of a clinical crown or abnormal clinical crowns, which are less suitable for alternative extraction methods, apical malformation and enlargement, lack of patient compliance for standing procedures, or concurrent pathology like pronounced local bone infection and/or sequestration, which would increase the risk of collateral damage and the overall morbidity of the procedure. However, once the surgeons became more skilled and experienced in performing partial coronectomies and intraoral sectioning, this reduced the need to resort to CAS in many cases with clinical crown abnormalities.

Unlike CAS repulsions, exodontia by means of intraoral sectioning, is, in most cases, successfully performed in standing horses. However, Leps and coworkers (34) recently reported surgery times of up to 4 hours and the need to convert to general anesthesia in 3/29 horses to complete the procedure. As long as patient safety is not compromised, a time-efficient approach, even when performed under general anesthesia, may offer advantages for the patient by reducing stress and the burden of enduring long procedures. Thus, particularly in selected cases where alternatives like MITSE, intraoral sectioning, or non-CAS-guided repulsion are considered challenging, potentially time-consuming, or associated with significant risks, CAS repulsion may offer a valuable and controlled approach.

A lateral buccotomy or dental alveolar transcortical osteotomy and buccotomy approach is another valid alternative, especially when apical malformation and enlargement makes repulsion more difficult. While this approach provides a direct visual control for removing dental tissues, it causes maximal disruption of the overlying soft tissues and alveolar bone. Based on our experience, it is also more time-consuming. With the availability of CAS-guidance, we elected in most cases to resort to CAS repulsion over the more invasive and time-consuming lateral buccotomy approach, although CAS-guidance is helpful with either approach.

In our hands, and regardless of the approach, CAS proved extremely valuable to complement advanced exodontia techniques that require optimal intraoperative orientation, including minimally invasive screw extraction and repulsion techniques. Challenging cheek tooth extractions are associated with a considerable risk for complications, especially if repulsion becomes necessary (1, 4, 35). This is frequently the result of surgical imprecision, particularly when considering the forces needed to expel the hypsodont teeth of equids and the close spatial arrangement of neighboring anatomical structures. By providing the operating surgeon with the best possible intraoperative orientation and real-time control over the instruments for critical steps of these procedures, CAS can help minimize collateral damage.

In addition, CAS facilitates minimally invasive and direct approaches, again helping to avoid important neurovascular structures in the approach, like the infraorbital or mandibular canal. In the present study, surgeons elected to use CAS most frequently for mandibular cheek tooth repulsions. We speculate that there are numerous reasons for this. First of all, most equine dentists and surgeons are more familiar and more comfortable in applying minimally invasive techniques for maxillary cheek tooth extractions to complement transoral exodontia in standing sedated equids. Furthermore, the more restricted access to mandibular cheek teeth with their narrower and more rectangular shaped alveolae, and the often limited information gained from conventional intraoperative 2D imaging in guiding minimally invasive techniques, such as MITSE or intraoral sectioning, might explain the overrepresentation of cases where mandibular cheek teeth were extracted with CAS and under general anesthesia.

The preoperative 3D imaging data, which is an integral part and essential for CAS, not only guides the procedure but also facilitates the early detection of anatomical anomalies or peculiarities. When combined with real-time intraoperative control—particularly the navigation of the punch with respect to orientation and penetration depth—this enables consistent alignment with the tooth’s long axis and early identification of any deviation in instrument positioning. This level of precision is especially critical when applying repulsion forces to mandibular cheek teeth and the mandible, a structure characterized by a thin cross-section and teeth with long reserve crowns.

Iatrogenically induced focal disruption of the mandibular cortex with a small diameter pin occurred in case 9, which was a 120 kg pony. The small dental punch abruptly slipped off the apex and got entrapped between the tooth and alveolar bone, causing a fine fracture line that was detected on intraoperative CBCT imaging in the mandibular bone. In this particular case, slippage of the dental punch was immediately recognized and addressed by changing to a larger punch and accurately aligning it with the tooth. The surgeon must be aware of this problem irrespective of the technique used. A similar focal disruption of the alveolar bone in the maxillary arcade was noted in the control scan of case 15, most probably due to surgical imprecision or the use of a small punch. These two minor, self-limiting complications underscore the importance of thoroughly loosening the tooth prior to repulsion, as this significantly reduces the force required for extraction/repulsion. Pre-extraction tooth loosening is essential for successful exodontia and should be performed with meticulous care whenever feasible.

In 6 out of 16 cases, follow-up surgeries in standing horses were required to remove osseous, dental, and sequestrated bone fragments. Based on our experience, it seems unlikely that major fragments were overlooked during intraoperative CT evaluations. Instead, the delayed onset of these complications likely reflects ongoing bone demarcation or sequestration. With the exception of one horse lost to follow-up due to reasons unrelated to CAS repulsion, the remaining 14 cases experienced successful outcomes without signs of dental repulsion-related issues.

For the repulsion step itself, the authors recommend using larger dental punches of adequate size and profile on their tip that allow for proper engagement of the tooth root and minimize the risk of slippage. The dental punch of choice, which was used for most horses in this study has a square shaped head of 10 × 14 mm and a profile with a rhomboid pattern (Figure 6). However, this requires that a small osteotomy be performed in the mandibular bone to introduce the dental punch. Although this increases the surgical invasiveness, it can be argued that the osteotomy approach can be placed precisely with the help of CAS and kept to a size just large enough to accommodate the selected punch and other instrumentation required. The latter are drill bits, a high-speed surgical drill, or an oscillating saw.

The rapid image acquisition, the large bore and high maneuverability of its gantry, make the mobile CBCT unit used in this study the ideal imaging tool for intraoperative imaging, thus allowing repeated image acquisition to assess progress of the surgical procedure as needed. This goes without compromising the diagnostic sensitivity in detecting dental abnormalities, when compared with helical CT imaging (36). During pre- and intraoperative imaging, all personnel left the room to avoid radiation exposure. Concerning radiation protection of personnel, CAS is in accordance with the ALARA principle, and the exposure is reduced compared to the frequently repeated intraoperative radiographic imaging that is mandatory for most challenging dental surgeries. Undoubtedly, compared to 2D radiographic imaging, the cross-sectional CBCT images with multiplanar and 3D volumetric reconstructions and the real-time intraoperative feedback provided by the navigation, give the operating surgeon a more comprehensive appreciation of the underlying pathology and the best possible orientation.

One limitation of CAS technology in dental surgery is the necessity for surgeons to be well-versed in its operating principles and aware of potential pitfalls that may compromise surgical accuracy. Any movement of the patient tracker relative to the targeted anatomical structures inevitably results in reduced precision. Additional factors affecting accuracy in cranial CAS include issues such as malfunctioning infrared optical digitizers or camera systems, often caused by blood contamination on reflective markers. Fortunately, the equine head provides several easily identifiable surface landmarks, like the facial crest, which serve effectively as fiducials. When a drop in navigational accuracy is suspected, the surgeon can verify the alignment of the virtual guide with actual anatomy by referencing the tip of the navigated pointer or instrument. The availability of a navigation system and the CAS-preparedness of the hospital infrastructure and personnel are essential for its successful application in a clinical setting.

The most important limitations of this study are the retrospective design and the small number of cases, which did not allow for statistical analysis and identification of specific risk factors for complications. Horses presenting with external swelling, a draining tract, and/or apical malformation or enlargement appear to be overrepresented among those requiring additional surgical interventions in our study.

In the present study, we share our first clinical experiences with navigated surgery in the field of equine dental surgery in anesthetized horses. We conclude that CAS is a valuable technology that can be applied for challenging equine dentistry applications to complement and potentially facilitate exodontia. Specifically, CAS repulsion represents a valid option in selected cases where conventional (less invasive) techniques have failed, or if surgical planning shows that these techniques will lead to failure in extreme cases. The main features that make this technology so useful for dentistry applications include the excellent intra-operative orientation and control it provides over instrumentation and forces applied during surgery. Therefore, CAS has the potential to reduce patient morbidity, radiation exposure to personnel, and surgery time for selected equine dentistry cases.

## Data Availability

The original contributions presented in the study are included in the article/supplementary material, further inquiries can be directed to the corresponding author.
